# SpikeSegNet-a deep learning approach utilizing encoder-decoder network with hourglass for spike segmentation and counting in wheat plant from visual imaging

**DOI:** 10.1186/s13007-020-00582-9

**Published:** 2020-03-18

**Authors:** Tanuj Misra, Alka Arora, Sudeep Marwaha, Viswanathan Chinnusamy, Atmakuri Ramakrishna Rao, Rajni Jain, Rabi Narayan Sahoo, Mrinmoy Ray, Sudhir Kumar, Dhandapani Raju, Ranjeet Ranjan Jha, Aditya Nigam, Swati Goel

**Affiliations:** 1 ICAR-Indian Agricultural Statistics Research Institute (IASRI), Library Avenue, Pusa, New Delhi 110012 India; 2 ICAR-Indian Agricultural Research Institute, New Delhi, India; 3 ICAR-National Institute of Agricultural Economics and Policy Research, New Delhi, India; 4 Indian Institute of Technology, Mandi, Himachal Pradesh India

**Keywords:** Deep learning, Encoder-decoder deep network, Image analysis, Non-destructive plant phenotyping, Wheat spikes identification and count

## Abstract

**Background:**

High throughput non-destructive phenotyping is emerging as a significant approach for phenotyping germplasm and breeding populations for the identification of superior donors, elite lines, and QTLs. Detection and counting of spikes, the grain bearing organs of wheat, is critical for phenomics of a large set of germplasm and breeding lines in controlled and field conditions. It is also required for precision agriculture where the application of nitrogen, water, and other inputs at this critical stage is necessary. Further, counting of spikes is an important measure to determine yield. Digital image analysis and machine learning techniques play an essential role in non-destructive plant phenotyping analysis.

**Results:**

In this study, an approach based on computer vision, particularly object detection, to recognize and count the number of spikes of the wheat plant from the digital images is proposed. For spike identification, a novel deep-learning network, SpikeSegNet, has been developed by combining two proposed feature networks: Local Patch extraction Network (LPNet) and Global Mask refinement Network (GMRNet). In LPNet, the contextual and spatial features are learned at the local patch level. The output of LPNet is a segmented mask image, which is further refined at the global level using GMRNet. Visual (RGB) images of 200 wheat plants were captured using LemnaTec imaging system installed at Nanaji Deshmukh Plant Phenomics Centre, ICAR-IARI, New Delhi. The precision, accuracy, and robustness (F_1_ score) of the proposed approach for spike segmentation are found to be 99.93%, 99.91%, and 99.91%, respectively. For counting the number of spikes, “analyse particles”—function of imageJ was applied on the output image of the proposed SpikeSegNet model. For spike counting, the average precision, accuracy, and robustness are 99%, 95%, and 97%, respectively. SpikeSegNet approach is tested for robustness with illuminated image dataset, and no significant difference is observed in the segmentation performance.

**Conclusion:**

In this study, a new approach called as SpikeSegNet has been proposed based on combined digital image analysis and deep learning techniques. A dedicated deep learning approach has been developed to identify and count spikes in the wheat plants. The performance of the approach demonstrates that SpikeSegNet is an effective and robust approach for spike detection and counting. As detection and counting of wheat spikes are closely related to the crop yield, and the proposed approach is also non-destructive, it is a significant step forward in the area of non-destructive and high-throughput phenotyping of wheat.

## Background

Wheat is one of the 3 major cereals, cultivated since the beginning of agriculture to support approximately 30% of the world population [1]. It is grown every year in around 215 million hectares in the world. Nearly US $50 billion worth of wheat is traded globally on yearly basis. It is the first and foremost food crop of 2.5 billion people in 89 countries [1]. Urbanization and rising incomes are driving a rapid rise in global wheat consumption. It is predicted that by 2050, consumers will require 60% more wheat production than today [2]. Further, this challenge needs to be addressed in the face of climate change and the associated increase in abiotic stresses. Therefore, a genetic improvement in wheat for input use efficiency and climate resilience is the key to future food security. Among the two pillars of genetic improvement of crops, genotyping is done with greater ease and accuracy now, while the phenotyping remains a rate-limiting step in the establishment of phenotype-genotype relationships. To remove this bottleneck, non-destructive image-based phenomics is gaining momentum worldwide. Visual image is the most common and low-cost method of imaging system used in non-destructive high throughput phenotyping as well as crop management, where image-based methods can help in input management.

Spike or ear emergence is a critical phenological event in wheat development, as it is required for the application of nitrogen, water, and other critical inputs for crop production. Further, yield estimation in wheat has received significant research attention as it is an important primary food for a large proportion of the world’s population [3]. Since spike number is a key factor that determines the grain number per unit area and thus yield, counting of the number of spikes/ears is an important measure to determine the yield of the plant [4]. Therefore spike detection and counting are important for phenology based input management for crop production and assessing the crop yield. Counting of the number of spikes per plant or per unit area through naked-eye is a laborious and time-consuming process. Hence, non-destructive spike detection and counting by image analysis are needed as a fast alternative. On the other hand, computer vision, particularly object detection through digital image analysis and machine learning techniques, plays an essential role in non-destructive plant phenotyping. It is strongly argued that future trends in image-based plant phenotyping will be a combined effort of image processing and machine learning for feature extraction and data analysis [5]. Most recently, deep convolutional network, i.e., a branch of machine learning, is being successfully applied in the area of object detection and classification and it out-performed many of the classical machine learning approaches in many diverse domains, for example, pattern recognition [6], instance detection and segmentation [7], UNet: for biomedical image segmentation [8], SegNet: for segmentation [9], plant disease detection and diagnosis [10], classification of fruits and flowers of plants from field images [11]. The deep neural network employs the mapping of the input layer to the output layer over a series of stacked layers of nodes [12]. A wide range of deep learning architectures have been used in plant phenotyping like, LeNet architecture used in biotic stress identification in banana [13], AlexNet and GoogLeNet used in apple scab and black-rot detection [10], Inception-v3 and ImageNet architectures for cassava brown streak disease detection [14] and CaffeNet and ImageNet architectures used in biotic stress detection in pear, cherry peach [15].

In the recent past, a galaxy of researchers worked in the area of computer vision to detect and characterize objects, such as spikes and spikelets in the wheat plant. Morphological image processing has been used in measuring spike characteristics, such as awn number, awn length, and spike length in wheat [16]. Also, spike identification method was developed to measure seven characteristic parameters with the images of individual spikes based on back-propagation neural network using Hu moments [17]. Here, images of detached spikes were used, and hence these methods are seemed to be not suitable for high-throughput and non-destructive detection and counting of intact spikes from the whole plant. Recently, some techniques have been developed for automatic detection of heading and flowering stage in wheat plant by using Bag-of visual- words approach [18]. In this approach, SIFT algorithm was used for low level feature extraction and finally support vector machine classification technique was used to classify growth stages. Besides, the accuracy of the method for spike detection was 85% and 99% at flowering and late growth stage, respectively. Li et al. [19] proposed an approach to detect and characterize the geometric properties of spikes of a single wheat plant grown in a controlled environmental condition. They used color index method for plant segmentation and neural network method with Laws texture energy for spike identification with around 80% accuracy. In these studies, color and texture were mostly used to identify wheat spikes, which are not totally machine-dependent as manual intervention is required to define the texture and range of color intensity for the segmentation purpose. In this context, Pound et al. [20] developed a deep learning approach for localizing wheat spikes and spikelets with around 95% accuracy, and the plants were imaged in a small-purpose built chamber with uniform background. Hasan et al. [21] used the region-based Convolutional Neural Network (R-CNN) approach to detect, count and analyze wheat spikes in the field condition. In this study, we propose an alternative approach to detect and count the number of spikes. The approach involves two stages: (i) identification of spikes on the digital image of the wheat plant and (ii) counting of spikes. Identification of spikes on the digital image is a class of pixel-wise segmentation problem of objects. Convolutional encoder-decoder deep learning based networks, viz, UNet [8], SegNet [9] and PixISegNet [22] are successfully used for pixel-wise segmentation of objects in various sectors. In the present study, a novel deep-learning network, known as SpikeSegNet, has been proposed by considering “U-Net” convolutional architecture [8] for spike identification. SpikeSegNet is a combination of two proposed feature networks: Local Patch extraction Network (LPNet) and Global Mask refinement Network (GMRNet). In LPNet, the contextual and spatial features are learned at the local patch level. The output of LPNet is a segmented mask image that may contain some inaccurate segmentation of the object, and is further refined at the global level by GMRNet. For counting the number of spikes from the analyzed image, *“analyse particles”* function of imageJ [23] has been applied on the output image (binary/segmented mask image containing spike regions only) of the SpikeSegNet model. To fix the problem of over-counting and undercounting of spike number per plant, different side views of the plant have been considered. The major advantage of the proposed approach is that it provides an efficient non-destructive approach of spike identification as well as counting of spikes in wheat plant through digital image analysis and deep learning technique.

## Materials and methods

### Image acquisition

Wheat plants were grown in pots in the climate-controlled greenhouse in Nanaji Deshmukh Plant Phenomics Centre, ICAR-Indian Agricultural Research Institute, New Delhi, India. Names of the genotypes used in this study are given in Additional file 1. The single wheat plant was grown to maturity in the pot with recommended cultural practices. Images of the plants were taken by using 6576 × 4384 pixel RGB camera (LemnaTec GmbH, Aachen, Germany). Three different side view images (angles: 0°, 120°, 240°) of the plants were recorded using the automated turning and lifting unit present inside the imaging unit. The side views were considered, as it is hypothesized that the image from one direction cannot cover all the spikes of the plant; besides, it helps in increasing the data points corresponding to one plant. Only side views were taken as it provides more information than the top view [19]. A uniform background was maintained to increase the accuracy of separation between background and plant regions. Images were stored in PNG format. Imaging was done during the reproductive stage of the plant. After imaging, the number of spikes per plant was counted manually, which is used as ground truth value to validate the proposed approach.

### Dataset preparation

**Fig. 1:**
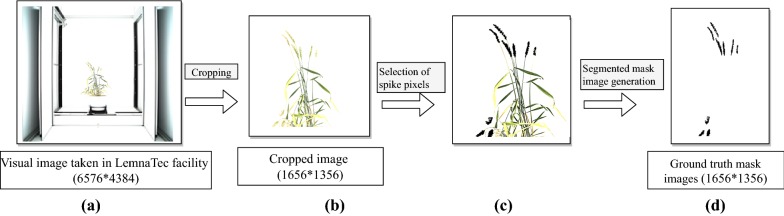
Dataset preparation: **a** LemnaTec image (of size 6576 × 4384) consists of not only the plant regions but also the chamber used in imaging; **b** the images are cropped (of size 1656 * 1356) to get only the region of interests; **c** spike pixels (x, y) are selected and previous color of the selected pixel is replaced by the fill_color; **d** segmented mask image is generated by setting the pixel value of fill_color as 0 (black) elsewhere, 255 (white)

**Fig. 2:**
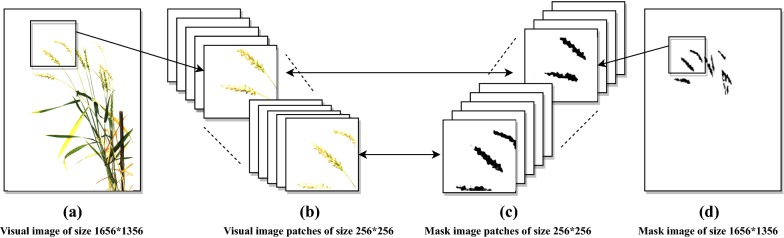
Patch generation: **a** visual images (of size 1656 * 1356) as well as **d** ground truth mask images (of size 1656 * 1356) are divided into 100 pixels overlapping patches [**b**, **c**] of size 256 * 256

The original size of the image was 6576 × 4384 pixels, which consist of not only the plant regions but also the chamber used in imaging, as shown in Fig. 1a. Hence, the images were cropped (of size 1656 * 1356) to get only the region of interest (plant regions) from the whole image, as shown in Fig. 1b. Visual images (RGB) and their corresponding ground-truth mask images with class labels (i.e., spike regions of the plant) were prepared manually [24] with the help of Photoshop software for training the network. In mask image, black pixels (pixel value = 0) represent the spike regions whereas white pixels (pixel value = 255) for the remaining portions of the corresponding visual image. Pseudocode of the segmented mask image preparation is given in Additional file 2. The output obtained from the running pseudocode is given in Fig. 1c, d. Visual images (of size 1656 * 1356) as well as ground truth mask images (of size 1656 * 1356) were divided into 100 pixel overlapping patches of size 256 * 256 for training the network (Fig. 2). Patches are nothing but the small overlapping portions of an image. Thus, from one image (of size 1656 * 1356), 180 patches (row wise 15 * column wise 12) were generated. Besides, training of network using patches also helped in learning local features more effectively than using the whole image [22].

### Proposed approach for detection and counting of spikes in a single plant

The proposed approach consists of two broad stages of analysis. First stage deals with identification or detection of spike regions from the whole wheat plant image and the second stage consists of counting of the number of spikes of the plant.

#### Proposed architecture of SpikeSegNet network for spike identification

The backbone of SpikeSegNet network is based on convolutional encoder-decoder deep-learning technique with hourglass, as a part of the bottleneck network. SpikeSegNet is a combination of two proposed feature networks:

A. Local Patch extraction Network (LPNet) and
B. Global Mask refinement Network (GMRNet).

**Fig. 3:**
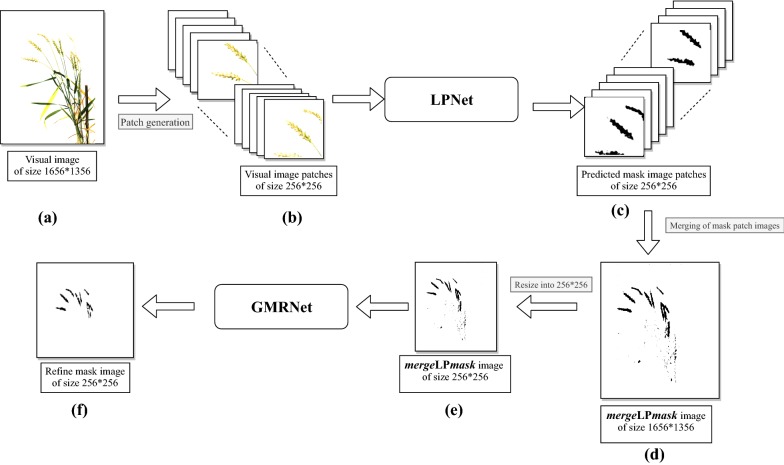
Flow diagram of SpikeSegNet: The network is developed for pixel-wise segmentation of objects (or spikes) from the wheat plant. SpikeSegNet is a combination of two proposed feature network namely Local Patch extraction Network (LPNet) and Global Mask refinement. Network (GMRNet). **a** The visual image of size 1656 * 1356 is divided into patches (**b**) of size 256 * 256 and fed into the LPNet network to extract contextual and spatial features at local patch level. Output of LPNet is segmented mask image patches (**c**) of size 256 * 256 which are then combined (*merge*LP*mask*) to generate the original mask image of size 1656 * 1356 (**d**); *merge*LP*mask* image may contain some sort of inaccurate segmentation of the object (or, spikes) and are refined at global level using GMRNet network; before passing through GMRNet, it is resized to 256 * 256 (**e**) to reduce the network complexity. The output of GMRNet network is nothing but the refined mask image (**f**) containing spike regions only

In LPNet, the contextual and spatial features are learned at the local patch level. The output of the LPNet is the segmented mask image patches of size 256 * 256, which are further combined to generate the original mask image (*merge*LP*mask*) of size 1656 * 1356. However, *merge*LP*mask* may contain some inaccurate segmentation of the object (or, spikes) and thus they are further refined at the global level using GMRNet. The overall flow-diagram of the proposed approach has been given in Fig. 3, and the detail of network architecture is discussed below:

##### A. LPNet

**Fig. 4:**
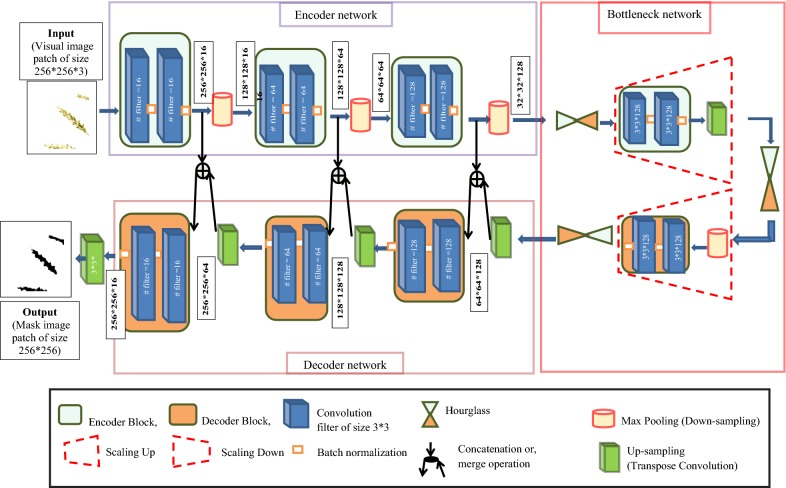
Architecture of LPNet network

The LPNet consists of Encoder, Decoder, along with Bottleneck network as shown in Fig. 4. Encoder takes input patch image to give feature map representation that holds the contextual and spatial information. The Decoder takes the information as input and produces corresponding segmentation masks as output [8, 12]. Skip connections [22] are formed between the encoder and the decoder and is also known as merge or concatenation operation, which helps in transferring the spatial information across the network for better localization of the segmentation masks. Through the skip connections, corresponding feature maps from the encoder before down-sampling (or, max-pooling) are concatenated with the corresponding feature maps of the decoder after up-sampling (or, transverse convolution). Bottleneck network in between encoder and decoder network has been introduced to compress the feature map representation for better segmentation results. The architecture of the proposed LPNet network consists of 3 encoder blocks, corresponding hierarchy of 3 decoder blocks, and 3 hourglasses between encoder-decoder as part of the bottleneck network. By introducing hourglass in the bottleneck, the segmentation network gives precise and contextually more confident segmentation mask. The numbers of encoder blocks, decoder blocks, and the hourglasses are estimated empirically to yield the best results with optimum performance. The details of encoder, decoder, and hourglass are discussed in follows.

Encoder network: The encoder network consists of 3 encoder blocks. The 1st encoder block takes the visual image patches (of size 256 * 256) as input and produces corresponding feature maps as output and forwarded it to the 2nd, followed by the 3rd encoder block for further feature extraction. Each encoder block contains a set of convolution layers to learn feature representation at different receptive fields [25], where each convolution layer applies a 3 * 3 filter followed by a non-linear activation function, namely Rectified Linear Unit (ReLU) [26]. Then these feature maps are batch normalized [27] to improve the performance and stability of the network followed by max-pooling with 2 * 2 window with stride 2 (non-overlapping) for sub-sampling or down-sampling the features by a factor of 2. Max-pooling operation using window size 2 * 2 or by sampling factor of 2, considered as standard [8, 10, 22, 28]. By choosing sample factor 2, the features are downsampled by half of its original size, and the aggregate features are extracted more effectively. Each encoder block is repeated with a varying filter depth of 16, 64, and 128 to encode the features. Square filter has been used as it is popularly used in various state-of-art methods [29] for the image of square shape. Details of each encoder block (i.e., input to each encoder block, number of convolution filters used with their sizes, the output of each encoder block, input, and output to the corresponding max-pool) are given in Table 1.

**Table 1:** Details of each encoder block and corresponding max-pool

Encoder block #	Input to encoder block	Convolution filter size	Number of convolution filter	Output of encoder block	Input to max-pool	Output to max-pool
Block 1	256 * 256 (patch image)	3 * 3	16	256 * 256 * 16	256 * 256 * 16	128 * 128 * 16
Block 2	128 * 128 * 16	3 * 3	64	128 * 128 * 64	128 * 128 * 64	64 * 64 * 64
Block 3	64 * 64 * 64	3 * 3	128	64 * 64 * 128	64 * 64 * 128	32 * 32 * 128

**Fig. 5:**
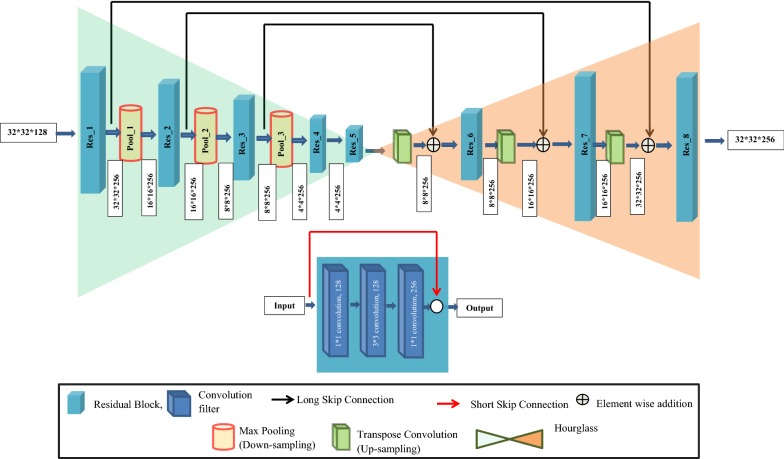
Architecture of Hourglass network (top) with residual block (bottom)

Bottleneck network: Output of the encoder network is passed as input to the bottleneck network. The bottleneck network consists of 3 hourglasses, scale up, and scale down operations. By introducing hourglass, it gives more confident segmentation result [8]. It is mainly due to the innate design of the hourglass network, which minimizes the feature map and captures the information by only concentrating on essential features. Scaling up and scaling down operation help in finding the relationship among aggregate features at different scales, which further helps to get the robust features [22]. Multiple hourglass networks (i.e., stacked hourglass) enhance the invariant features that are captured at various scale, viewpoint and occlusion very effectively to predict the segmentation mask of the image accurately [22]. But, more than one hourglass in the network will increase the network depth and performance may fall due to over-fitting. As there is a trade-off between the number of parameters and accuracy, we have chosen one scale up and one scale down operations along with 3 hourglasses empirically to get the best results with optimal parameters and optimum performance. The hourglass consists of two parts: Hourglass Encoder (Hg^E^) and Hourglass Decoder (Hg^D^). Each layer in Hg^E^/Hg^D^ is realized as layer Residual Module/Block. The Residual Module/Block consists of a 1 * 1 convolution of depth 128 followed by 3 * 3 convolution of depth 128 and then 1 * 1 convolution of depth 256 (Fig. 5). Each residual block again consists of skip connection that allows the flow of gradient and spatial information across the deep network and facilitates in solving the problem of vanishing gradient. In general, Encoder/Decoder network, after each max pool step, the output of the encoder block is concatenated with the corresponding decoder block. In hourglass network, instead of concatenating the layer of the encoder with that of the decoder, the layer is further convolved through residual block and then added element-wise to the corresponding layer of the decoder. Hg^E^ network receives the output from the encoder network and contains four residual modules in sequential order and Hg^D^ network contains 3 long skip connections to preserve the spatial information along with four residual modules in sequential order (Fig. 5). The Input and output of each hourglass is given in Table 2.

**Table 2:** Input and output of each hourglass

Hourglass	Input	Output	After scale up	After scale down
Hourglass 1	32 * 32 * 128	32 * 32 * 128	64 * 64 * 128	_
Hourglass 2	64 * 64 * 128	64 * 64 * 128	_	32 * 32 * 128
Hourglass 3	32 * 32 * 128	32 * 32 * 128	_	–

“_” indicates the corresponding operation has not been done

Decoder network: In decoder, the output from the 3^rd^ hourglass (32 * 32 * 128) is up-sampled using a 3*3 transpose convolution with padding 1 and stride 1. Then the resulting feature map (of size 64 * 64 * 128) got concatenated with the corresponding encoder feature map. The concatenated feature map (of size 64 * 64 * 256) is then passed to two, 3 * 3 convolution layers (padding 1 and stride 1) followed by ReLU activation and Batch Normalization and is repeated with filters having varied channel depths of 128, 64 and 16 as opposite to the encoder blocks. Details of each decoder block (i.e., input to the each decoder block, number of convolution filter used with their sizes, output of each decoder block, input and output to the corresponding transpose convolutional layer) are given in Table 3. The output of the final decoder has been fed into 3 * 3 * 1 convolution layer with “softmax” activation function [30] to classify the object (i.e., spike) at the patch level. The output of LPNet is a predicted mask image patch of size 256 * 256 corresponding to the input patch image (i.e., visual image patch of size 256 * 256), as shown in Fig. 4.

**Table 3:** Details of each decoder block and corresponding transpose convolutional layer

Decoder block #	Input to transpose convolution	Output of transpose convolution	Input to decoder block	Convolution filter size	Number of convolution filter	Output of decoder block
Block 1	32 * 32 * 128	64 * 64 * 128	64 * 64 * 128	3 * 3	128	64 * 64 * 128
Block 2	64 * 64 * 128	128 * 128 * 64	128 * 128 * 64	3 * 3	64	128 * 128 * 64
Block 3	128 * 128 * 64	256 * 256 * 64	256 * 256 * 64	3 * 3	16	256 * 256 * 16

##### B. GMRNet

**Fig. 6:**
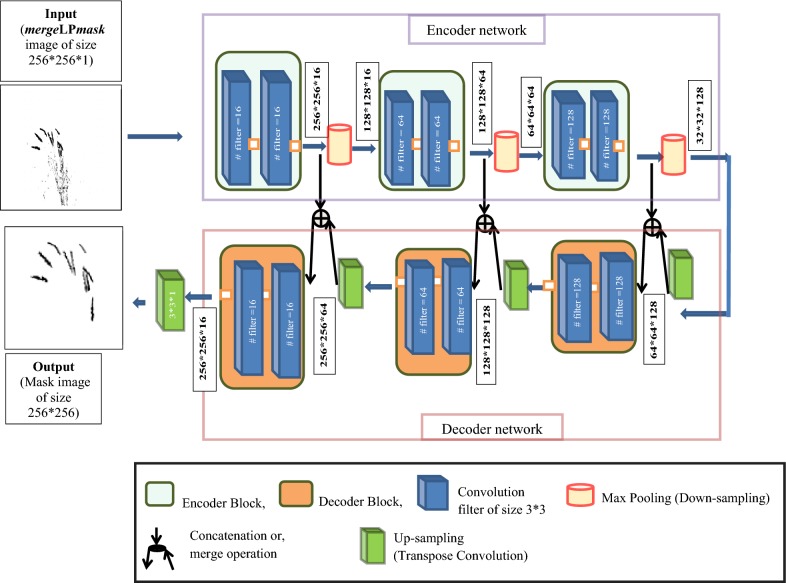
Architecture of GMRNet network

The GMRNet consists of 3 encoder blocks and the corresponding hierarchy of 3 decoder blocks. The architecture has also been developed by taking inspiration from UNet architecture [8]. The architecture of GMRNet is the same as the architecture of LPNet without hourglass network. The input of GMRNet is *merge*LP*mask* image of size 256 * 256. Outline of the architecture is given in Fig. 6. The inner-structure, input, output and hyper-parameter of the each encoder and decoder block is the same as given in Tables 1 and 3 respectively.

#### Approach for counting number of spikes in a single plant

The *“analyse particles”* function of imageJ [23], which implements flood-fill technique [31], has been used in counting the number of spikes per plant. The flood-fill technique employs object count by growing through similar pixel regions from the starting pixel. The *“analyse particles”* function counts and measures (pixel area) objects in binary or threshold images. The visual images of single plant from three direction side views (0°, 120°, 240°) have been used as input to the proposed SpikeSegNet model and then the *“analyse particles”* function was applied on the predicted output mask images (i.e., refined mask image of size 256 * 256) to count number of objects (spikes) in a single plant. Three side views are considered as image from one direction cannot cover all the spikes of a plant.

### Model development and performance measurement

To develop/train the SpikeSegNet model, dataset consisting of images taken from 3 directions of 200 plants was considered. The dataset was subsequently bifurcated randomly into training set and validation set with 85% and 15% of the total plants respectively. For developing SpikeSegNet, both the network models (LPNet and GMRNet) have been trained on Linux operating system with 32 GB RAM and NVIDIA GeForce GTX 1080 Ti graphics card (with memory 11 GB).

Training of LPNet: The training dataset contains randomly selected 85% images of the total plants (i.e., images of 170 plants out of 200). As 3 direction images corresponding to one plant have been considered, the dataset consists of 510 images (i.e., 170 plant * 3 direction image) of size 1656 * 1356. The network has been trained by using visual image patches. Each of the 510 visual images (of size 1656 * 1356) has been divided into 100 pixel overlapping patches (of size 256 * 256), and from one image, 180 patches (row wise 15 * column wise 12) were generated. Hence, total 91,800 (510 image * 180 patches) patches have been used for training the network. Training strategy of the network involves multi-stage modular training [22] where each module is trained independently and then merged to form a single network, which is then trained in end-to-end fashion in the final stage. The model was developed by using “Adam” optimizer [32] with a learning rate of 0.0005 to update the weights. The network was trained for 200 epochs with batch size of 32 images (each of 256 * 256) as per the system constraints. “Binary cross-entropy” [22] was used as loss function to predict binary class label (i.e., spikes and non-spikes) at the patch level. It is the most commonly used loss function in the image segmentation to compute pixel-wise cross entropy. It examines each pixel individually and compares its binary class predictions (either 0/1; in this context, spikes region or not) to the ground truth (or, segmented ground truth mask image consisting of spike regions only). Cross entropy loss evaluates the class predictions for each pixel individually and averages it over all the pixels. Therefore, each pixel contributes uniformly to the overall objective loss function. Feature maps (i.e., output of applying the filters to the input image or, another feature map) are given in Additional file 3. It shows the sample output feature maps after applying the encoder network, hourglass network, and the decoder network in the LPNet.

Training of GMRNet: The output of LPNet is a predicted mask image patch of size 256 * 256 corresponding to the input patch image (i.e., visual image patches of size 256 * 256), as shown in Fig. 3. Therefore, from 91,800 (= 510 image * 180 patches) visual image patches (i.e., training dataset), the same number of mask image patches will be generated. The predicted mask image patches (of size 256 * 256) are then combined to construct *merge*LP*mask* image (of size 1656 * 1356), as discussed in the earlier section. Hence, 180 predicted mask image patches corresponding to the single visual image of size 1656 * 1356 are combined to construct a single *merge*LP*mask* image. In this way, from 91,800 mask image patches (of size 256 * 256), 510 (= 91,800 patches/180 patches) *merge*LP*mask* images (of size 1656 * 1356) have been generated. These images have been resized into 256 * 256 (to reduce the network complexity) and used in training GMRNet using the same system configuration used in training the LPNet network. Similar to LPNet, this network has also been developed by using “Adam” optimizer [32] with a learning rate of 0.0005 to update the weights. The network has also been trained for 200 epochs with batch size of 32 images (each of 256 * 256) and “Binary cross-entropy” loss function has been used to predict binary class label (i.e., spikes and non-spikes).

#### Performance measures for identification of spikes

The segmentation performance of the SpikeSegNet in the identification/detection of spikes was analyzed on the validation image dataset. The dataset was then passed through LPNet followed by GMRNet using the step-wise procedure as outlined in the flow diagram (Fig. 3) and the final output mask image (i.e., predicted refined mask image of size 256 * 256) (Fig. 3f) was compared with the ground-truth segmented mask image prepared using the pseudocode mentioned in Additional file 2 under “Dataset preparation” section. The pixel-wise segmentation performance was evaluated by the performance parameters [Type I Classification Error (E1), Type II Classification Error (E2), Jaccard Index (JI)] discussed in the literature [22, 33–35]. Following performance parameters were used for measuring the segmentation performance and are defined as follows:

- True positive (TP_D): # pixels correctly classified as spikes pixels.
- True Negative (TN_D): # pixels correctly classified as non-spikes pixels.
- False Positive (FP_D): # non-spikes pixels classified as spikes pixels.
- False Negative (FN_D): # spikes pixels classified as non- spikes pixels.

Precision, Recall, F-measure and Accuracy are defined as:

- 1 $$ \begin{aligned}\Pr ecision &= \frac{TP\_D}{TP\_D + FP\_D}\ {\text{denotes the }}\% \\ &\quad{\text{ of detected pixels are actually spikes}}\end{aligned} $$
- 2 $$ \begin{aligned}\text{Re} call &= \frac{TP\_D}{TP\_D + FN\_D}\ {\text{measures}}, \, \%\\ &\quad {\text{ of actually spikes spike pixels are detected}}\end{aligned} $$
- 3 $$ \begin{aligned}Accuracy & = \frac{TP\_D + TN\_D}{TP\_D + TN\_D + FP\_D + FN\_D}\\&\quad {\text{ indicates the performance of the proposed approach}}\end{aligned} $$
- 4 $${\text{F}}_{ 1} Score = 2\frac{precision*recall}{precision + recall} {\text{\, measures robustness of the proposed network in detecting or identifying spikes}}$$

Comparative analysis for spike identification with illuminated images: A comparative analysis was conducted with the illuminated images to test the robustness of the proposed approach for spike segmentation. The validation image dataset was artificially illuminated by applying Gamma correction ($\gamma$) at different levels from darker to the brighter illumination. Gamma correction is a non-linear operation used in encoding and decoding luminance values in the image [36]. We have considered gamma values 0.1 to 2.5, from darker to brighter, as the level of illumination may not go beyond that in the field conditions. The illuminated image dataset was passed into the SpikeSegNet network as input, and the step-wise operations were done to produce the predicted mask image as output. Subsequently, the performance parameters [E_1_, E_2_, JI, Eq. (1), Eq. (2), Eq. (3), Eq. (4)] are computed at different levels of illumination to analyze the segmentation performance of the proposed approach.

Comparative analysis for spike identification with the existing approach: The performance of SpikeSegNet has also been compared with the approach given by Li et al. [19]. Broadly, the latter approach involves 3 steps. In the first step, the color index method was applied for plant segmentation. Several color indices [(R–G), (G–B), (G−B)/(R−G), (2G−R–B) and (3G−2.4R–B)] were used based on digital number (DN) of each band [red (R), green (G) and blue (B)]. In the second step, spikes were detected by applying the neural network-based (NN) method with Laws texture on the output image of the first step, and the resultant output was further improved by removing noise using area and height threshold in the third step. The performance of spike detection was measured by manually checking all the spikes in the image. Accuracy was measured by taking the ratio of the number of correct spikes detected with the total number of spikes. For comparative study, we have applied the 3-step procedure of Li et al. [19] on our validation image dataset (case 1) as well as applied our proposed approach, SpikeSegNet, on the sample dataset provided by Li et al. [19] at the website (link: https://sourceforge.net/projects/spike-detection/) (case 2). For comparing the pixel-wise segmentation performance in Case 2, the ground truth mask image has been prepared based on their dataset by using the pseudocode mentioned Additional file 2 under “Dataset preparation” section, and the segmentation performance has been measured by the performance metrics.

#### Performance metrics for counting number of spikes

Performance of the spike count approach was evaluated on the validation image dataset (i.e., randomly selected 15% of the total plant) by the procedure given in “Approach for counting number of spikes in a single plant” section. The output obtained i.e., spike count using imageJ was manually compared with the ground truth value. The performance of the developed model in counting number of spike was evaluated on the basis of precision, recall, accuracy and the F_1_ score based on true positive (TP_C), false positive (FP_C), true negative (TN_C), and false negative (FN_C) which are defined as follows:

- $TP\_C =$ number of objects correctly classify as spike.
- $FP\_C =$ number of objects incorrectly classify as spike (i.e., leaf, background) or overlapping spikes (connected objects).
- $FN\_C =$ number of actual spikes that are not visible in any of the side image.
- $TN\_C =$ is always’zero’ in this binary classification problem as background is not determined for object detection.

Precision, recall, accuracy and F_1_ score were measured by using the above Eqs. (1–4) where, Precision measures the % of detected objects which are actually spikes, Recall depicts the % of actually spikes that have been detected among the ground truth, Accuracy measures the performance of the approach and F_1_ score measures robustness of the approach.

## Results

**Fig. 7:**
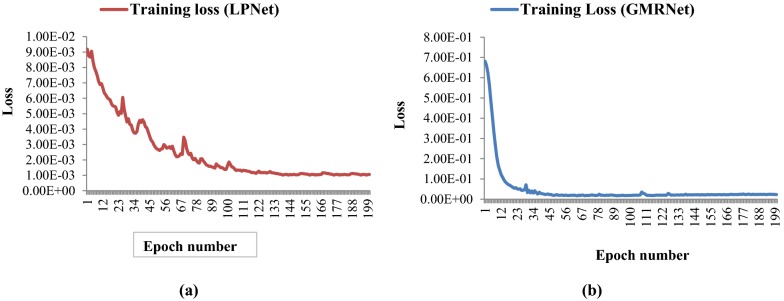
**a** Training loss in LPNet is plateaued around 135 epochs and incase of **b** GMRNet it is around 45 epochs

In this experiment, a total of 600 images of 200 plants from 3 directions were recorded. Image dataset of randomly selected 85% of the total plants (i.e., 510 images of 170 plants) were used in developing the SpikeSegNet network model to identify the spike regions on the digital image of the plant. 510 RGB images and their corresponding ground-truth segmented mask images were used in training the proposed network. In the first phase of training, LPNet was trained at patch level using 91,800 patches of size 256 * 256. The efficiency of the training model was analyzed by the training loss and error-rate at each epoch during the learning stage of the network. Although the training was done for 200 epochs, a remarkable decrease in the loss was measured at each epoch of training. The loss was initially high, and a plateau in the decrease was found around 135 epochs (Fig. 7a). In the second phase of training, GMRNet was trained using 510 *merge*LP*mask* images. Although the training was done for 200 epochs, a plateau in the decrease was found around 45 epochs (Fig. 7b).

**Fig. 8:**
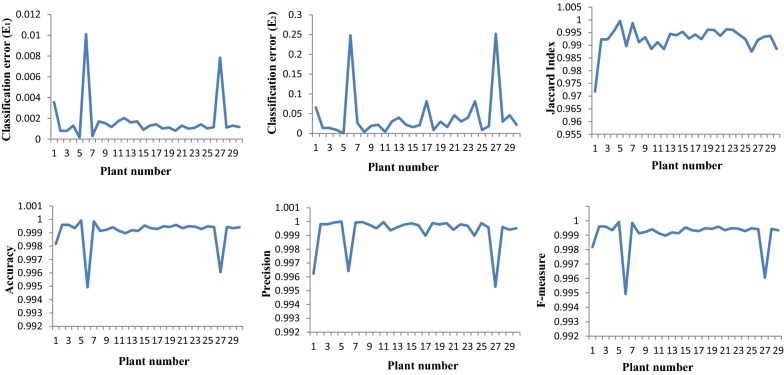
Graphical representation of E1, E2, Jaccard Index (JI), Accuracy, Precision, and F-measure of 30 different plants

The performance analysis of the proposed segmentation network, SpikeSegNet, for identification of spikes was tested on the validation dataset consisting of randomly selected 15% of the total plants (i.e., 90 images from 3 side-directions of 30 plants each). The segmentation performance is measured by different performance parameters (E_1_, E_2_, Jaccard Index (JI), Accuracy, Precision, Recall, and F-measure) and the average values of these parameters are shown in Table 4. The graphical representation of E_1_, E_2_, Jaccard Index (JI), Accuracy, Precision, Recall, and F-measure of 30 plants are shown in Fig. 8. Here, for each plant, average measurement of the metrics of 3 direction images (0°, 120°, 240°) are presented.

**Table 4:** Performance analysis of SpikeSegNet approach on our validation dataset

E_1_	E_2_	JI	Accuracy	Precision	Recall	F-measure
0.0016	0.0487	0.9982	0.9991	0.9993	0.9989	0.9991

### Segmentation performance in spike identification with illuminated images

**Fig. 9:**
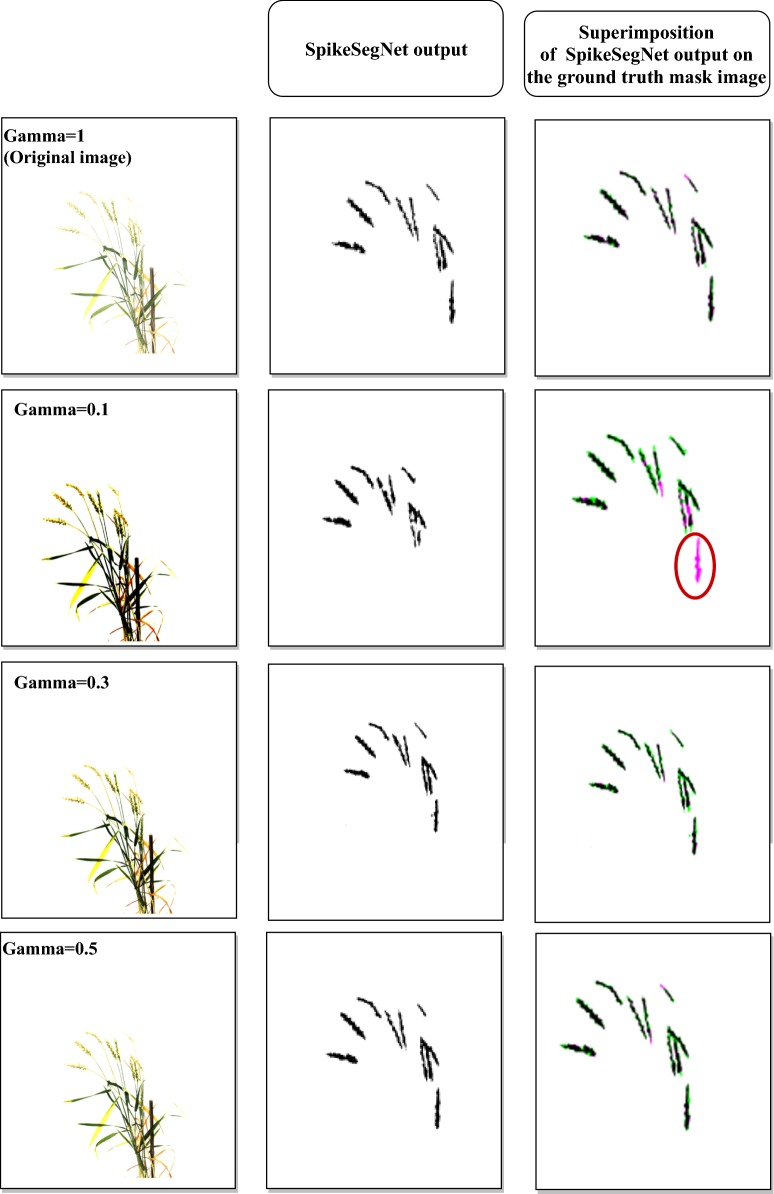

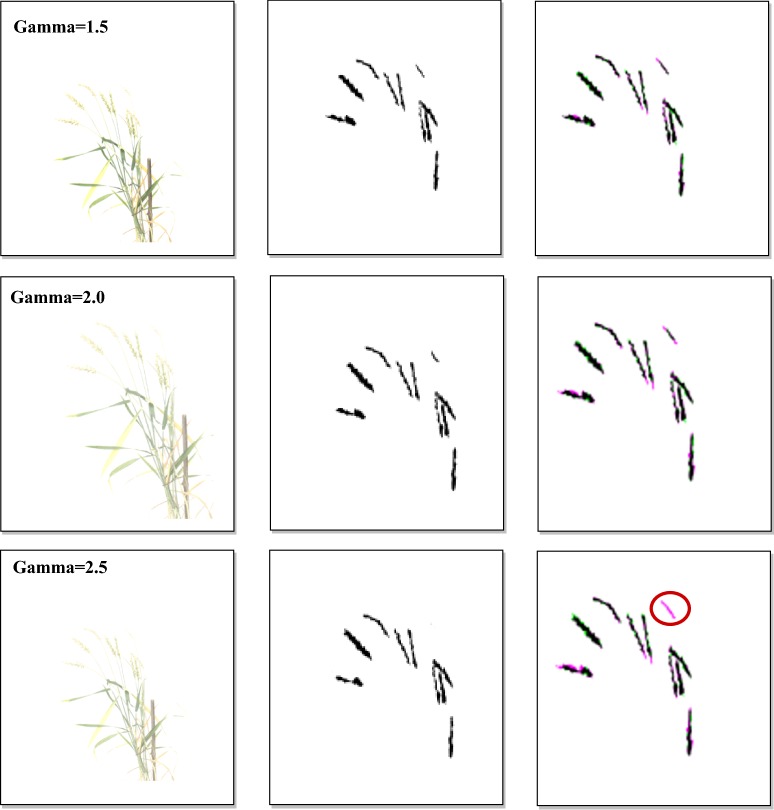
Segmentation performance with different levels of illuminated images: First column shows the illuminated images at different gamma values; Second column represents the output images after applying SpikeSegNet approach; For visually analyzing the pixel count error, output of SpikeSegNet at different level of illumination is superimposed on the ground truth segmented mask image (manually prepared).The colored pixel (pink and green) represents the wrongly classified pixels where pink indicates that actual spike pixels are not identified and the green pixels indicate that non-spike pixels are misclassified as spike pixels. The circles on the figure represent the spike pixels which are not detected

Performance of SpikeSegNet has been tested on the illuminated image dataset consisting of various levels of illumination from darker to brighter. After applying $\gamma$ operation on the same validation image dataset at different levels [0.1, 0.3, 0.5, 1.5, 2.0, 2.5], the outputs are shown in first column of Fig. 9. Sample output after applying the SpikeSegNet model at different levels of illumination is shown in second column of Fig. 9. For visual analysis of pixel count error, output of SpikeSegNet at different levels of illumination were superimposed on the ground truth segmented mask image (manually prepared) and shown in the third column of Fig. 9. The colored pixels (pink and green) represent the wrongly classified pixels where pink indicates that actual spike pixels are not identified, and the green pixels indicate that non-spike pixels are misclassified as spike pixels. The circles on the Fig. 9 represent the spike pixels that are not detected. The performance analysis is given in Table 5. It is observed that SpikeSegNet has a stronger ability to perform spike segmentation at different levels of illumination (from Table 5 and Fig. 9).

**Table 5:** Performance analysis of SpikeSegNet approach on illuminated dataset

	Gamma 0.1	Gamma 0.3	Gamma 0.5	Gamma 1 (original image)	Gamma 1.5	Gamma 2.0	Gamma 2.5
Classification error rate (E_1_)	0.005349223	0.003051249	0.002396139	0.001693726	0.00177917	0.0020579	0.002359009
Classification error rate (E_2_)	0.083304137	0.042303843	0.040107991	0.04874738	0.06317883	0.08736489	0.108331881
Average_Precision	0.998094286	0.999318633	0.999408703	0.999325313	0.99911935	0.99879521	0.998452183
Average_Recall	0.996521902	0.997607927	0.998178134	0.99896944	0.99908922	0.99913291	0.998812372
Average_F_1_measure	0.997302422	0.998461999	0.998792781	0.999147219	0.99910416	0.99896389	0.998812369
Average_Accuracy	0.997302421	0.998462003	0.998792786	0.999147223	0.99910416	0.9989639	0.998812372
Average_Jaccard_Index_for_Spike_detection:	0.994627456	0.99693175	0.997591056	0.998298182	0.99821199	0.99793177	0.997629168

### Segmentation performance for spike identification with the existing approach

**Fig. 10:**
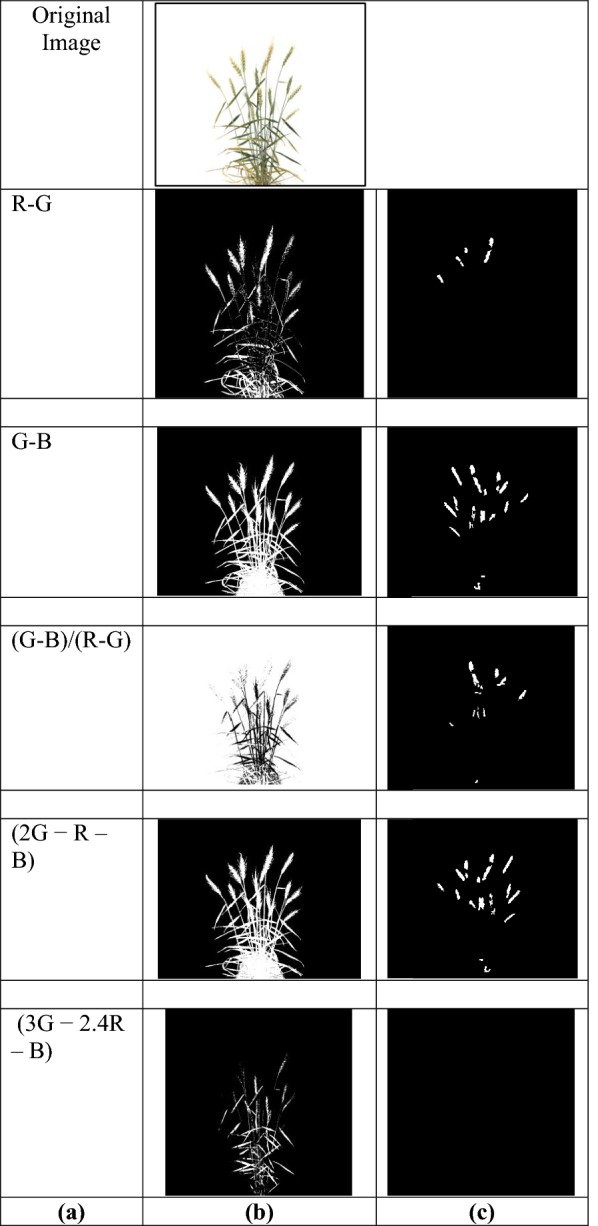
Comparative analysis with the approach given by Li et al. [19] (case 1): **a** shows the various color indices used in plant segmentation; segmentation results (step 1) displayed in **b**; **c** represents the outcomes after applying neural networks with Laws texture energy on the segmented images (step 2) and by removing noise using area and height threshold (step 3)

**Fig. 11:**
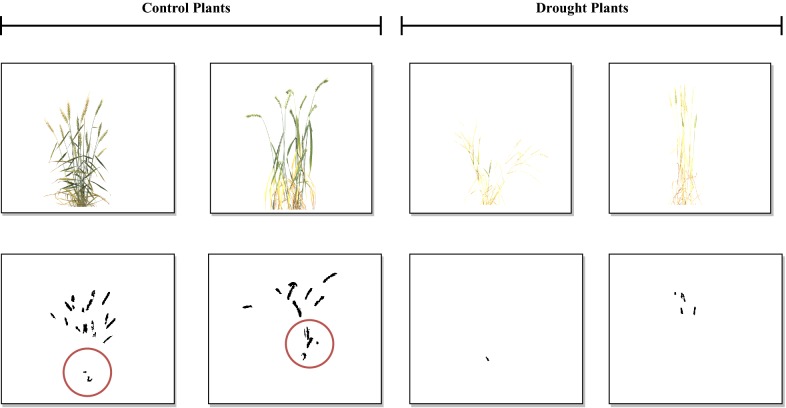
Sample output after applying (2 g − r–b) color indices with the NN approach (Li et al. [19]) on our validation dataset consists of randomly selected control as well as drought plants. The circle shows the wrongly classified spike pixels

**Fig. 12:**
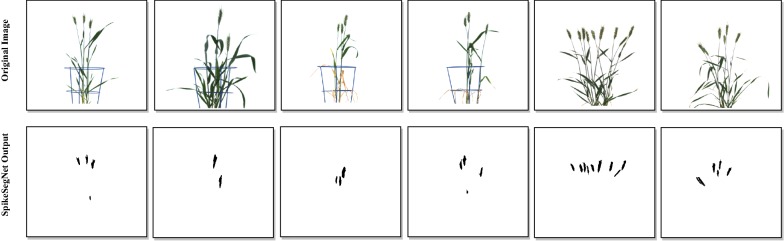
(Case 2) Output of SpikeSegNet on the sample dataset provided in the website (link: https://sourceforge.net/projects/spike-detection/)

For comparing the performance of SpikeSegNet with the existing approach, we have applied Li’s approach [19], on our validation image dataset (case 1) (Fig. 10). Figure 10a shows the various color indices used in plant segmentation; segmentation results (step 1) displayed in Fig. 10b, c represents the outcome after applying neural networks with Laws texture energy on the segmented images (step 2) and by removing noise using area and height threshold (step 3). The validation dataset consists of randomly selected images of well-irrigated and drought stressed plants. The sample output after applying Li’s approach on the validation dataset is given in Fig. 11, which reflects that the segmentation performance is far better in well-irrigated plants than that of drought stressed plants. The circles in the Fig. 11 show wrongly classified spike pixels. It was found that (2g– r–b)-color index out-performs all the color indices with an accuracy of around 81% while considering the well-watered plants. Besides, SpikeSegNet, was also applied on the sample dataset provided by Li et al. [19] (case 2) and the output is shown in Fig. 12. The pixel-wise segmentation performance is given in Table 6.

**Table 6:** Performance analysis of SpikeSegNet approach on the sample dataset provided on the website (link: https://sourceforge.net/projects/spike-detection/)

E_1_	E_2_	JI	Accuracy	Precision	Recall	F-measure
0.00172	0.0488	0.9982	0.9991	0.9984	0.9989	0.9991

### Performance analysis in counting number of spikes per plant

**Fig. 13:**
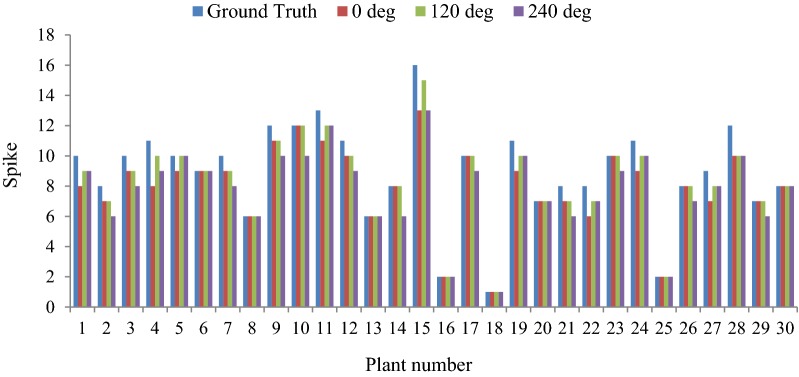
Comparative study of spike count with three side views (0°, 120° and 240°) and the ground truth

For counting the number of spikes in wheat plant, the developed model has been tested on the same validation dataset as well as the approach described in “Approach for counting number of spikes in a single plant” section. It is observed that, the maximum spike count obtained from the images of three directions (0°, 120°, 240°) of the single plant is very closely associated with the ground truth spike count (Fig. 13). Hence, the image with maximum spike count was used to compare and evaluate the performance of the said approach for spike counting. In order to validate the counting approach, resultant output mask images were superimposed over the original images (RGB images). The precision, accuracy and F_1_ score corresponding to the 30 plants are represented in Table 5. The average precision, accuracy and F_1_ score are observed that 99%, 95% and 97%, respectively.

## Discussion

The performance of spike identification is calculated at pixel level, and it can be observed from the value of classification error (E_1_), that, on an average only 105 pixels were classified wrongly among 65,536 (256 * 256) pixels of one image. Accuracy of the developed model is close to 100% and spikes are detected with an average precision and recall of 99.93% and 99.89%, respectively. The precision value reflects that 99.93% of the detected pixels are actually spikes, whereas recall value reflects that 99.89% of actual spike pixels are detected among the ground truth spike pixels using the developed network. Average F_1_ score reveals that the proposed network is 99.91% robust in identifying/detecting spikes from whole plant images. We have tested the robustness of SpikeSegNet approach with diversified illuminated image dataset as well as with another approach given by Li et al. [19]. The performances obtained are discussed as belows.

### SpikeSegNet vs illuminated image dataset

**Fig. 14:**
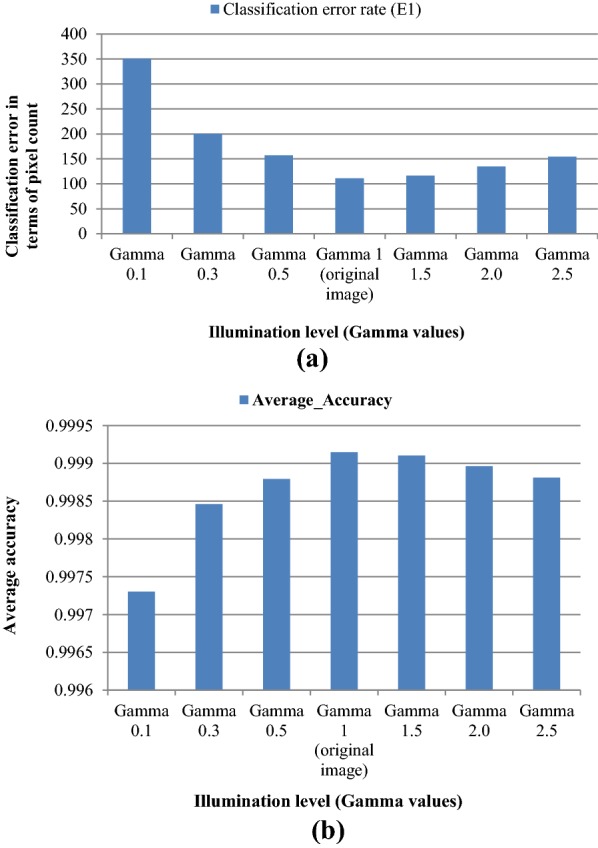
Graphical representation of **a** Classification error (E1) in terms of pixel count and **b** average accuracy in the mentioned illumination level

As the performance of spike identification was calculated at pixel level, it can be observed from the value of the classification/segmentation error (E_1_) given in Table 5, that on an average only 351 (0.00535%) pixels at gamma 0.1, 200 (0.00305%) pixels at gamma 0.3, 157 (0.00239%) pixels at gamma 0.5, 116 (0.0017%) pixels at gamma 1.5, 135 (0.0027%) pixels at gamma 2.0 and 155 (0.00235%) pixels at gamma 2.5 were classified wrongly out of 65,536 (256 * 256) pixels of one image. The values of E_1_ at different illumination levels have also been graphically represented in Fig. 14a, which reflects that classification/segmentation error is minimum at gamma = 1 (original image), and it is gradually increasing when gamma value is either decreasing or increasing from 1. To check the consistency of SpikeSegNet in terms of classification error at different levels of illumination, we have also computed Least Significant Difference (LSD) at 5% level of significance and the result revealed that classification error is comparatively higher at gamma 0.1 and is at per (i.e., no significant difference) at gamma 0.3 to 2.5. The accuracies are almost consistent from gamma value ranging from 0.3 to 2.0 (Table 5 and Fig. 14b). Although the effect of illumination level (gamma values ranges from 0.1 to 2.5) on the original image has a significant difference in image quality, as shown in the first column of Fig. 9, the proposed approach has very well-addressed such type of issues. Thus, the SpikeSegNet approach can adapt to the different illumination levels and also provides a high segmentation performance.

### SpikeSegNet vs Li’s approach

For comparing the performance of SpikeSegNet with Li’s approach, we have considered two cases. In case 1, from Fig. 10, it can be easily observed that, performance of NN approach with Laws texture energy [19] is better in the color indices (G–B), (G–B)/(R–G) and (2G–R–B) as compared to (R–G) and (3G–2.4R–B) indices. Li’s approach considered (3G–2.4R–B)-index for plant segmentation, it might be due to the presence of several factors *like* the background of imaging, light condition presence during imaging, accessories present in the imaging chamber, etc. From Fig. 11, it is reflected that the segmentation performance of Li’s approach is far better in the well-irrigated plants than in the drought-stressed plants, which may be due to the consideration of well-irrigated plants as well as color based features for developing the approach. Hence, Li’s [19] approach may have limited application for analysis of stress plants with leaf senescence and drying and plants near to the maturity where leaf and spike often have similar color. In case 2, it can be observed from Fig. 12 that the spikes presented on the sample image dataset are well detected by SpikeSegNet approach. The pixel-wise segmentation performance is given in Table 6, and the value of E_1_ is on an average only 0.00172% per image. We have obtained an average accuracy of 99.91%, with average precision and recall of 99.93% and 99.89%, respectively, which is almost similar to the performance results (Table 4) obtained on our validation dataset. It is because of, SpikeSegNet approach has been trained on patch level for training local as well as global features from the images taken from plants subjected to diverse treatments (from irrigated to drought stressed).

**Fig. 15:**
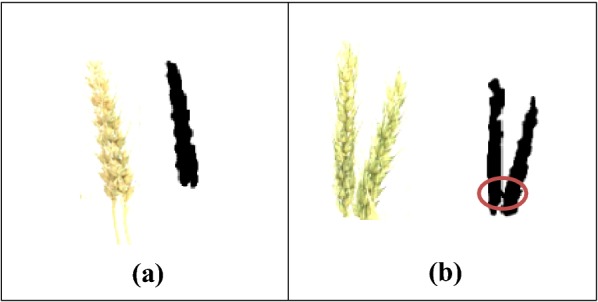
**a** Overlapping problem, **b** object connecting problem

Although the proposed SpikeSegNet model achieved 99.91% accuracy in identifying spikes (Table 5), but the counting accuracy is about 95% (Table 7). This may be due to undercounting of spikes that overlap each other and object linking (or connecting) problem (Fig. 15). As flood-fill technique employs object count by growing through similar pixel regions from the starting pixel the multiple objects are treated as if they are linked together and counted as one object.

**Table 7:** Outcome of the approach of spike counting on the test images of 30 plants

Image no	Ground truth	Predicted using model	TP	FP	FN	Precision	Accuracy	F1 score
1	10	9	9	0	0	1.00	1.00	1.00
2	8	7	7	0	0	1.00	1.00	1.00
3	10	9	8	0	0	1.00	1.00	1.00
4	11	10	10	0	1	1.00	0.91	0.95
5	10	10	10	0	0	1.00	1.00	1.00
6	9	9	8	1	0	0.89	0.89	0.94
7	10	9	9	0	0	1.00	1.00	1.00
8	6	6	6	0	0	1.00	1.00	1.00
9	12	11	10	0	1	1.00	0.91	0.95
10	12	12	11	0	1	1.00	0.92	0.96
11	13	12	10	0	1	1.00	0.91	0.95
12	11	10	9	0	1	1.00	0.90	0.95
13	6	6	6	0	0	1.00	1.00	1.00
14	8	8	8	0	0	1.00	1.00	1.00
15	16	15	13	2	1	0.87	0.81	0.90
16	2	2	2	0	0	1.00	1.00	1.00
17	10	10	10	0	0	1.00	1.00	1.00
18	1	1	1	0	0	1.00	1.00	1.00
19	11	10	10	0	0	1.00	1.00	1.00
20	7	7	7	0	0	1.00	1.00	1.00
21	8	7	7	0	1	1.00	0.88	0.93
22	8	7	7	0	1	1.00	0.88	0.93
23	10	10	10	0	0	1.00	1.00	1.00
24	11	10	10	0	1	1.00	0.91	0.95
25	2	2	2	0	0	1.00	1.00	1.00
26	8	8	7	0	0	1.00	1.00	1.00
27	9	8	7	0	1	1.00	0.88	0.93
28	12	10	10	0	2	1.00	0.83	0.91
29	7	7	7	0	0	1.00	1.00	1.00
30	8	8	7	1	0	0.88	0.88	0.93
Average						0.99	0.95	0.97

The previous studies [16–19] involve more laborious and complex procedures to develop image processing pipeline by manually defining texture and color intensity ranges in a particular situation to detect and characterize spikes in wheat plants. Bi et al. [16] and Lv [17] achieved ~ 87% accuracy for spike identification, but, they have used destructive method for taking images. In this counter, [18] and [19] proposed a non-destructive approach for spike identification. But, it involves a hectic job of image pre-processing as well as manually defined textures as input to the machine learning model to identify the spikes. In the recent trend, it has been seen that computer vision, particularly, object detection plays an important role in non-destructive plant phenotyping through digital image analysis and deep-learning technique for automatic detection of spikes in wheat [20, 21] as well as in other valuable sectors [10, 11, 13–15] in agriculture. In this context, a novel approach has been presented in this study based on combined effort of digital image analysis and deep learning techniques which involve identification as well as counting of spikes from the digital images of whole wheat plant. It’s not only eliminating the tedious job of image pre-processing but also, manual intervention of defining the features for the particular task. The main beauty of the approach is to automatically detect and learn from the features to produce the desire output. For designing and building the deep learning network, some iterative optimization is needed in taking decision about the number and size of the network layers and tuning the hyper-parameters. Although the above considerations are essential for training the network, it is less cumbersome and robust approach for spike detection.

## Conclusion

Detecting and counting of wheat spikes in a non-destructive and high throughput manner is a challenging task in the area of plant phenotyping. In this study, a new approach SpikeSegNet has been presented based on combined digital image analysis and deep learning techniques to identify and count spikes in the wheat plant. The proposed deep learning network achieved an accuracy of 99.91% in spike identification, whereas 95% average accuracy is achieved in spike counting. The approach is tested with different illumination levels for its robustness in the field conditions. SpikeSegNet approach can adapt to the different illumination levels and also provides a good segmentation performance. Further non-destructive detection of ear emergence will be useful in the automation of phenology based treatments in automated phenomics facilities, nutrient/water application in precision agriculture. As counting of wheat spikes is closely related to the crop yield and the proposed approach is efficient in identifying wheat spike, it is a significant step forward in the area of non-destructive and high-throughput phenotyping of wheat.

## Supplementary information

**Additional file 1.** Names of the genotypes used for the study.

**Additional file 2.** Pseudocode of the segmented mask image preparation.

**Additional file 3.** Feature maps (i.e., the output of applying the filters to the input image or, another feature map).

## Data Availability

The datasets used and/or analysed during the current study are available from the corresponding author on reasonable request.

## Abbreviations

E_1_: Type I error
Pix_Err: Pixel-wise classification error
E_2_: Type II error
FPR: False positives rates
FNR: False negatives rates
JI: Jaccard index
