# Supplementary material for: SpikeSegNet-a deep learning approach utilizing encoder-decoder network with hourglass for spike segmentation and counting in wheat plant from visual imaging
Source: Plant Methods. 2020 Mar 18;16:40. doi: 10.1186/s13007-020-00582-9 (PMC7079463; doi:10.1186/s13007-020-00582-9)
Supplement: Supplementary file 2 — Additional file 2. Pseudocode of the segmented mask image preparation. [file 13007_2020_582_MOESM2_ESM.docx]

**Pseudocode:** **Segmented Mask Image Preparation [I, I^M^, (x, y), fill_color, prev_color]**

**Input**: Visual image (**I**) of size p*q

**Output**: Corresponding mask image (**I^M^**) of size p*q

**(x, y)**: Selected pixel on **I**

**fill_color**: Filling/Painting color (black color) of the selected pixel (x, y) by flood-fill approach [Asundi & Wensen (1998)]

**prev_color**: Previous color of the selected pixel (x, y)

*Step* 1: Select pixels (x, y) belonging to wheat spikes using “wand tool” [Lehr et al. (1999)] manually for visual image I of size p*q // p=number of rows, q=number of columns

*Step* 2: Fill/Paint each spike pixel by “fill_color” using 4-connected flood-fill approach [Firstly, the selected pixel (x, y) is filled by fill_color by replacing the prev_color and then the four neighboring points: right (x, y+1), left (x, y-1), above (x-1, y) and below (x+1, y) corresponding to the (x, y) pixels are filled correspondingly]:

floodfill (x, y, fill_color, prev_color,p,q):

begin

SetSpikePixel (x, y, fill_color)

floodfill (x+1, y, fill_color, prev_color)

floodfill (x-1, y, fill_color, prev_color)

floodfill (x, y+1, fill_color, prev_color)

floodfill (x, y-1, fill_color, prev_color)

end

*Step* 3: Generate mask image containing spike regions (I^M^): If color of the selected pixel (x, y) of visual image (I) is fill_color (black), then set the pixel value=0, otherwise 255 (white/background)

if I (x, y) == fill_color

then, set I (x, y) =0

else, set I (x, y) = 255

*Step* 4: Return I^M^

**Additional file 1:** Pseudocode of segmented mask image preparation
