# Supplementary figures and images for: SpikeSegNet-a deep learning approach utilizing encoder-decoder network with hourglass for spike segmentation and counting in wheat plant from visual imaging
Source: Plant Methods. 2020 Mar 18;16:40. doi: 10.1186/s13007-020-00582-9 (PMC7079463; doi:10.1186/s13007-020-00582-9)

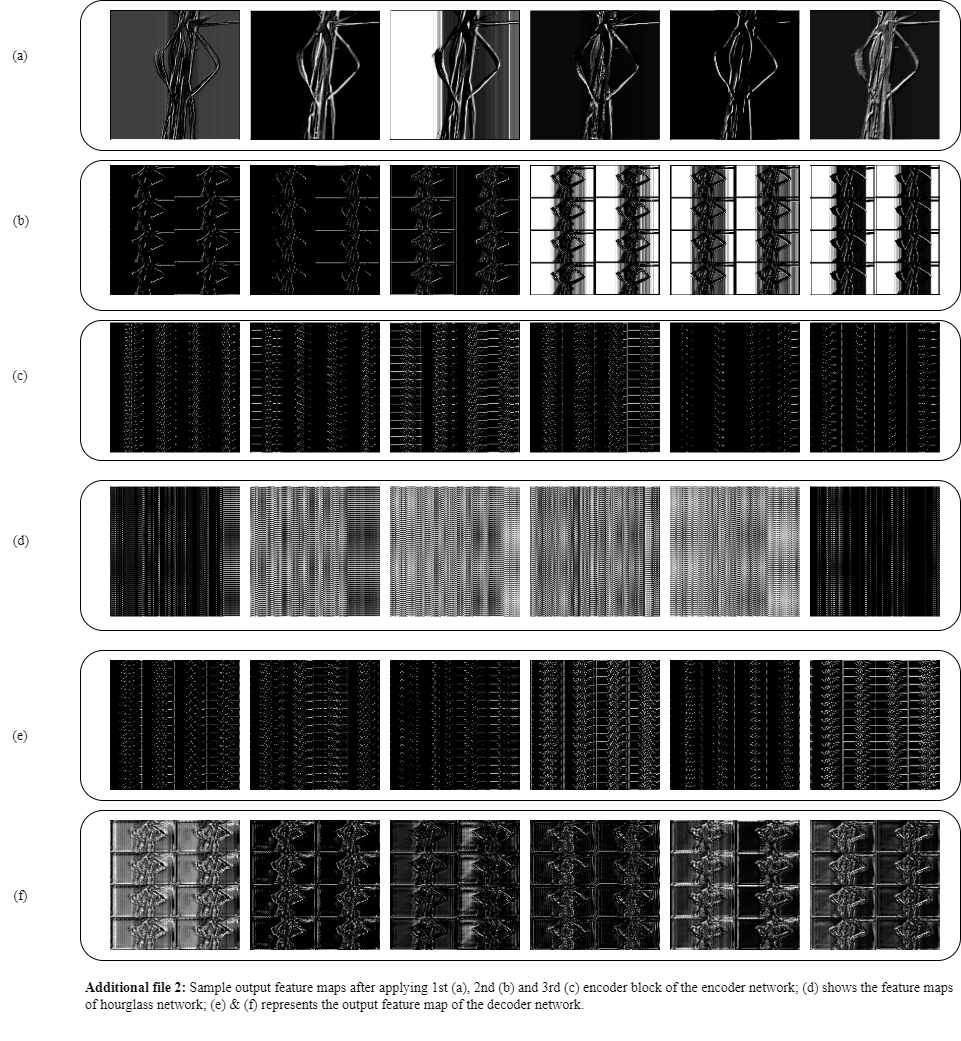

Supplement: Supplementary file 3 — Additional file 3. Feature maps (i.e., the output of applying the filters to the input image or, another feature map). [file 13007_2020_582_MOESM3_ESM.png]
